# Notalgia Paresthetica: Cervical Spine Disease and Neuropathic Pruritus

**DOI:** 10.7759/cureus.12975

**Published:** 2021-01-28

**Authors:** Ayesha Akram

**Affiliations:** 1 Internal Medicine, Rawalpindi Medical University, Rawalpindi, PAK

**Keywords:** notalgia paresthetica, cervical spondylosis, enigmatic link

## Abstract

Notalgia paresthetica (NP) is a dermatologic condition with predominant, primarily left unilateral pruritus and hyperpigmentation that typically occurs on the upper and middle back. The etiology remains largely elusive. A 57-year-old female with a history of neck pain presented with refractory NP since six months. Through diagnostic x-ray, cervical degenerative changes were discovered at the C5-C6 level, and she was prescribed a course of cervical traction. The cervical theory of NP is presented and is supported with x-ray findings in this case.

## Introduction

Notalgia paresthetica (NP) is a cutaneous sensory neuropathy that predominantly affects females, with onset at middle age or older [1,2]. Although it is common, patients underestimate their symptoms, and physicians present an inertia to consider the possibility of NP, and far fewer know about the neuropathic itch. Doubtless, many cases go largely unrecognized, underdiagnosed, or overlooked in the routine clinical practice [3,4].

Pruritus is the overwhelming clinical symptom in the majority of patients [1,5]. A left-sided and posterior location matches well with the location of NP; almost always, NP is unilateral [1]. Hyperpigmentation in the affected area often results from scratching itchy, desensate skin [1]. Along with the pruritus, patients may also experience burning, tingling, coldness, hyperesthesia, hypoesthesia, numbness, or nerve pain in the area where pruritus appeared [6].

Neuropathic itch and pain are signaling abnormalities - the causative lesion may be half a meter away from where the symptoms are felt, in a nerve, nerve root, spinal cord, or the brain. Spinal nerves and roots are vulnerable to compression as they exit the spinal cord, and they can be chronically compressed by narrowing of the bony foramina. The cause of NP may remain clouded if advances in imaging do not refine diagnosis. The features of NP in a 57-year-old woman, with x-ray-proven cervical spondylosis, are presented in this article.

## Case presentation

A 57-year-old female presented to the dermatology outpatient department with a six-month history of refractory, recurrent bouts of localized pruritus in otherwise healthy skin, most characteristic along the superior left posterior thoracic wall. She also identified chronic mild neck pain, which she rated as 3/10 on a zero to 10 numerical pain rating scale. The sensation of itch was restricted to the skin only, did not exhibit any day-to-night variability, and commonly outlasted any inciting stimulus. It was a potent trigger of scratching even during times of inattention causing much discomfort and nuisance, although this provided only fleeting relief. The patient first underwent an evaluation by a dermatologist four months back. A diagnosis of NP was made on physical inspection only. A trial of topical clobevate (corticosteroid) twice a day (BD), topical nerisone (corticosteroid) BD, topical lidocaine (local anesthetic) three times daily, and oral desloratadine (histamine H1 receptor antagonist) 5 mg BD provided little relief to the itch. Her medical history was significant for peptic ulcer disease, postmenopausal lumbar osteoporosis, and obesity. It was negative for eczema, contact dermatitis, medication side effects, infections affecting the skin, and disorders associated with chronic pruritus. Her other medications include ibandronate (bisphosphonate) IV once every three months for lumbar osteoporosis and famotidine 20 mg BD for peptic ulcer disease.

**Figure 1:**
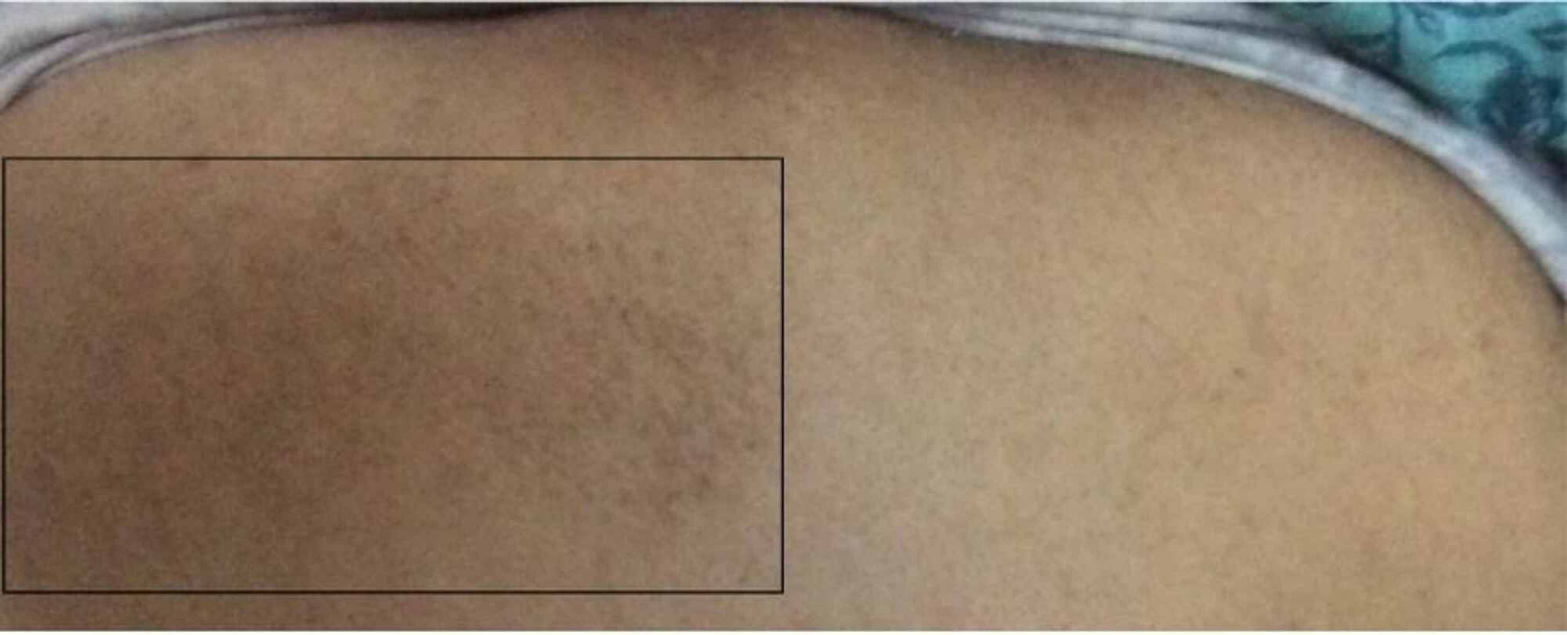
Refractory notalgia paresthetica upper back in a female The relevant area of the back is only included. The symptomatic area mainly medial to the patient’s left scapula is visible as a hyperpigmented patch (within the box).

Performed laboratory tests, slightly on or out of normal limit, are included: hemoglobin A1c 6.3%, fasting plasma glucose 5.7 mmol/L, a bone mineral density of 0.752 g/cm^2^, and a T-score of -2.7 at the L1-L4 level. Findings on physical examination of the patient’s back include skin findings of a unilateral, ill-defined, nonindurated tan, or hyperpigmented patch on the upper back, as shown in Figure 1. The area of pruritus demonstrated mild sensory alterations to light touch and pinprick but no evidence of dermatitis, induration, warmth, or swelling. There was minimally decreased range of motion in the neck and mild tenderness particularly in the cervical level four (C4)-cervical level seven (C7) area, but no painful muscle spasms, motor weakness (winged scapula, for instance), or signs of another peripheral neuropathy. Shoulder movements and range of motion were normal.

**Figure 2:**
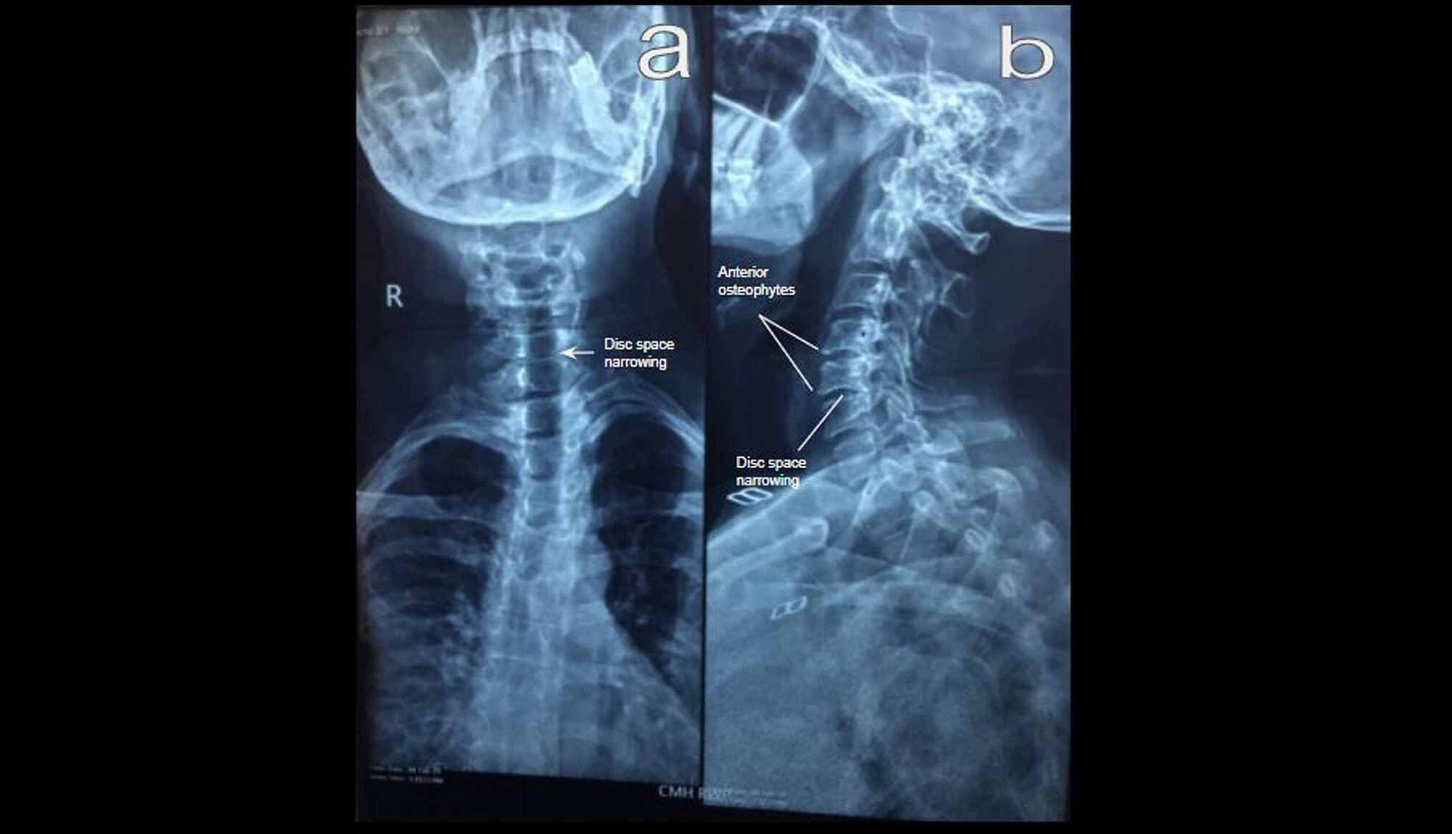
Cervical spondylosis on anteroposterior (a) and lateral (b) views of x-ray - multilevel degenerative changes from C4 through C6 X-ray findings of degeneration including reduced intervertebral space at the Cv5-Cv6 level (labeled) and anterior marginal lipping (labeled) at a few levels.

It was decided to perform a basic cervical spinal x-ray to investigate the anatomic area of insisting pruritus and for establishing a diagnosis (Figure 2). The thoracic x-ray was clear.

This patient had tried all: from orals to topicals to self-help strategies to halt the errant itch’s interference. NP did not respond to traditional pain relievers or traditional antipruritic drugs. Additionally, topical agents for neuropathic pain like capsaicin and lidocaine had been ineffective too. Treatment was thus directed at the underlying neck pathology. She was diagnosed with NP; prescribed a cervical soft collar, spinal manipulation, and a six-week course of mechanical traction of the C4-C6 vertebrae; and advised follow-up after two months. She was educated on association with cervical spine disease, use of cervical soft collar, and proper neck posture. A patient-reported outcome is warranted at follow-up to fully evaluate the effect of conservative (noninvasive) spinal treatment as first-line therapy for NP. She declined cryolipolysis, but it is reasonable to counsel her to consider cryolipolysis an option at the subsequent visit if this does not work.

## Discussion

NP has a dermatologic presentation, but there is no primary dermatologic pathology. Rather, it can be taken as a cutaneous sign of underlying spinal nerve impingement at the C4-C6 level due to the degenerative cervical spine [5]. The spine causes radicular compression (pinched nerve) when distorted by an overt structural lesion, e.g., spondylosis and disc herniation [6]. Spondylosis is an age-related degeneration of the vertebral bodies, discs, and joints. Disc space decreases, and joint stress results in osteophyte formation, which slowly narrows the neural foramina leading to nerve root compression [7].

The telltale symptom of NP in this patient - insisting pruritus at a predominantly left-sided, posterior thoracic location, extending over four to six dermatomes, mainly medial to the scapula - prompted specific workup in order to confirm or rule out the diagnostic hypothesis. Cervical spine disease was otherwise mildly clinically evident in this patient. An x-ray consistent with degenerative changes and osteophyte formation is an evidence to support the central cervical etiology of NP. Symptoms of cervical spondylosis are typically slowly progressive rather than those of rapid onset, as seen in this patient. Even though a cervical magnetic resonance imaging (MRI) scan was not done, an osteophyte compressing the afferent sensory or exiting C5 or C6 nerve root is the most likely etiology in this case.

The skin is richly innervated with the small unmyelinated (C-fibers) and thinly myelinated (A-delta fibers) axons that transmit itch and pain sensations [2,8]. Findings so far suggest lesions anywhere along the peripheral nervous system or central nervous system that damage itch-transducing, conducting, or processing neurons can cause neuropathic itch [2]. A fragmentary understanding is gleaned of the anatomical pathways that mediate NP from a comprehensive review. One theory of a cervical source for NP depends on the albeit rare, cutaneous sensory location of the dorsal scapular nerve [9]. The dorsal scapular nerve arises from C5 in 70%, C4 in 22%, and C6 in 8% of individuals [10] and can go on to innervate a small area of skin corresponding to the fifth and sixth thoracic vertebrae [9]. Another likely assumption is discussed; damage to peripheral somatosensory axons of the long thoracic nerve can also cause neuropathic itch [9]. This reasoning to reconcile the cervical theory with the dermatomal pattern seen in NP may only fail on the premise that these are primarily motor nerves and rarely responsible for any cutaneous sensation.

An article shows that if you study 100 people in their 40s walking around without any back/neck problems, about half will have degenerative changes in the thoracic spine and 90 will have degenerative changes in the cervical spine. The changes included decreased signal intensity of the intervertebral discs, disc protrusion, anterior compression, and disc space narrowing [11]. An Ortho-Derm tandem is at the forefront of the concept that NP is caused by nerve entrapment due to degenerative changes in vertebrae. In this study, they demonstrate a correlation between vertebral pathology and NP dermatomes in seven out of 10 cases. Evaluation of these intriguing findings is hindered by the lack of case details and imaging pictures [12]. Evidence to support the central cervical theory includes a case of NP with confirmed cervicospinal disease at C4-C6 [5]. A classic case of NP has been described in which the patient’s symptoms persisted for two years despite multiple physicians and treatments. She had a left-sided neural foraminal stenosis at the C7-T1 level demonstrated on computed tomography (CT) scan. After recognizing her NP was spinal in etiology, she was cured by cervical traction [13]. In a pathological study of 12 cases with NP, nine patients scanned using x-rays were found to have spinal disorders. Four out of six patients got a reduction in symptoms after spinal physiotherapy [14].

## Conclusions

Degenerative cervical spine disease spurred neuropathic pruritus. The itch was not a sign of inflammation, allergy, or an underlying disorder. A dermatologist should first examine the patient to look for the cause of pruritus in the symptomatic area, but neurological causes, particularly in the presence of musculoskeletal (even if it’s just neck pain) or neurologic symptoms, should be considered for otherwise-unexplained itch.

## References

[1] Ellis C (2013). Notalgia paresthetica: the unreachable itch. Dermatol Pract Concept.

[2] Robbins BA, Rayi A, Ferrer-Bruker SJ (2020). Notalgia paresthetica. StatPearls [Internet].

[3] Howard M, Sahhar L, Andrews F, Bergman R, Gin D (2018). Notalgia paresthetica: a review for dermatologists. Int J Dermatol.

[4] Šitum M, Kolić M, Franceschi N, Pećina M (2018). Notalgia paresthetica. Acta Clin Croat.

[5] Alai NN, Skinner HB, Nabili ST, Jeffes E, Shahrokni S, Saemi AM (2010). Notalgia paresthetica associated with cervical spinal stenosis and cervicothoracic disk disease at C4 through C7. Cutis.

[6] Cohen PR (2017). Notalgia paresthetica: a novel approach to treatment with cryolipolysis. Cureus.

[7] Kuo DT, Tadi P (2020). Cervical spondylosis. StatPearls [Internet].

[8] Oaklander AL, Siegel SM (2005). Cutaneous innervation: form and function. J Am Acad Dermatol.

[9] Muir B (2017). Dorsal scapular nerve neuropathy: a narrative review of the literature. J Can Chiropr Assoc.

[10] Nguyen VH, Liu HH, Rosales A, Reeves R (2016). A cadaveric investigation of the dorsal scapular nerve. Anat Res Int.

[11] Matsumoto M, Okada E, Ichihara D (2010). Age-related changes of thoracic and cervical intervertebral discs in asymptomatic subjects. Spine (Phila Pa 1976).

[12] Şavk E, Şavk Ö, Bolukbasi O, Ccedilulhaci N, Dikicioğlu E, Karaman G, Şendur N (2000). Notalgia paresthetica: a study on pathogenesis. Int J Dermatol.

[13] Low R, Swanson LA, Swanson DL (2017). Notalgia paresthetica relieved by cervical traction. J Am Board Fam Med.

[14] Raison-Peyron N, Meunier L, Acevedo M, Meynadier J (1999). Notalgia paresthetica: clinical, physiopathological and therapeutic aspects. A study of 12 cases. J Eur Acad Dermatol Venereol.

